# Early Psychosis Intervention: A Culturally Adaptive Clinical Guide

**DOI:** 10.1192/pb.bp.114.047985

**Published:** 2015-02

**Authors:** Roger Ho

**Figure:**
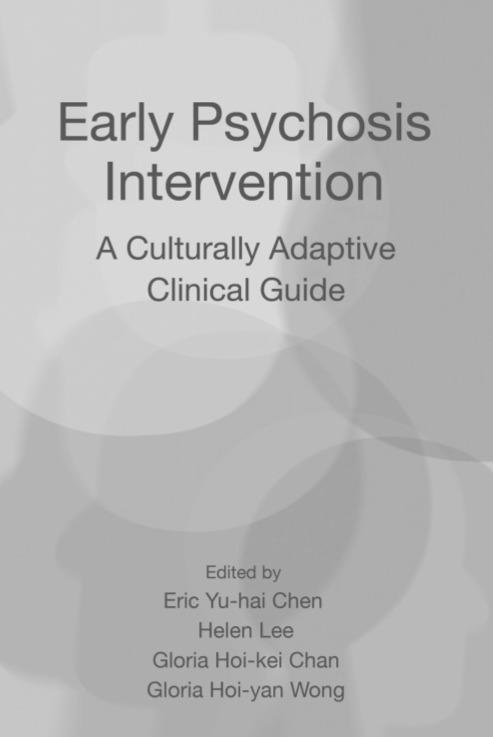


This book is written by multidisciplinary pioneers in early psychosis intervention in South-East Asia and is a product of two decades of development in this rapidly growing region, a cultural mosaic. In the foreword, Professor Patrick McGorry highlights that the essence of such intervention is to bring maximum recovery for young people with psychotic experiences.

The book is unique and attempts to connect early psychosis with transcultural psychiatry. The first part describes service structures of early psychosis programmes in Hong Kong, Singapore, Japan and Korea. The authors define early psychosis afresh by introducing the culturally adaptive translation in Chinese, *sijueshitiao*, meaning imbalance of thinking and perception. The second part discusses the cultural issues in management of early psychosis. The authors tactfully compare and contrast different Asian beliefs of mental illness including Islam (unbalanced lifestyle), Hindu (bad karma), Buddhism and Taoism (attacks by ghosts) and Christianity (demonic possession). The local cultural beliefs may increase duration of untreated illness because patients and families often consult their traditional healers instead of medical practitioners.

The authors present interesting data on public misconceptions about psychosis. They highlight salient points in early psychosis treatment such as recommended dose of each antipsychotic drug, topics to be covered in peer support groups, strategies for family work and medication adherence. I personally found the chapter illustrating the state-of-art information technology and database design very interesting.

I highly recommend this book to mental health professionals who are keen to establish early psychosis intervention services in other parts of Asia, Africa and South America. The authors carefully insert clinical vignettes throughout the book and enrich its clinical relevance. Mental health professionals working for well-established early psychosis intervention programmes may find the culturally adaptive strategies helpful in their clinical practice. In the near future, I hope Professor Eric Chen and his colleagues may consider writing a book on the neurobiology of early psychosis.

